# High beta rhythm amplitude in olfactory learning signs a well-consolidated and non-flexible behavioral state

**DOI:** 10.1038/s41598-019-56340-y

**Published:** 2019-12-30

**Authors:** Nicolas Fourcaud-Trocmé, Laura Lefèvre, Samuel Garcia, Belkacem Messaoudi, Nathalie Buonviso

**Affiliations:** 1 Lyon Neuroscience Research Center, Inserm U 1028, CNRS UMR 5292, University Lyon 1, Bron, 69675 France; 2 Medical Research Council Brain Network Dynamics Unit, University of Oxford, OX1 3TH Oxford, United Kingdom

**Keywords:** Neural circuits, Olfactory cortex

## Abstract

Beta rhythm (15–30 Hz) is a major candidate underlying long-range communication in the brain. In olfactory tasks, beta activity is strongly modulated by learning but its condition of expression and the network(s) responsible for its generation are unclear. Here we analyzed the emergence of beta activity in local field potentials recorded from olfactory, sensorimotor and limbic structures of rats performing an olfactory task. Rats performed successively simple discrimination, rule transfer, memory recall tests and contingency reversal. Beta rhythm amplitude progressively increased over learning in most recorded areas. Beta amplitude reduced to baseline when new odors were introduced, but remained high during memory recall. Intra-session analysis showed that even expert rats required several trials to reach a good performance level, with beta rhythm amplitude increasing in parallel. Notably, at the beginning of the reversal task, beta amplitude remained high while performance was low and, in all tested animals, beta amplitude decreased before rats were able to learn the new contingencies. Connectivity analysis showed that beta activity was highly coherent between all structures where it was expressed. Overall, our results suggest that beta rhythm is expressed in a highly coherent network when context learning - including both odors and reward - is consolidated and signals behavioral inflexibility.

## Introduction

Beta rhythm has been suggested as a major candidate supporting long range information transfer through functional coupling of neurons across distant brain regions^1^. In the sensorimotor cortex, it may reflect cortical idling^2,3^. Engel and Fries (2010) suggested beta activity could be related to signaling the status quo^4^, *i.e*. the maintenance of the sensorimotor set during the next processing step. In the olfactory system, beta activity is a major rhythm^5^. However, disparities in the literature have hindered attempts of a unifying hypothesis, either for the condition of its expression or for determining the network(s) involved in its generation.

In anesthetized rats, beta expression is promoted by odorant stimulation with low vapor pressure molecules^6^. In awake animals, however, it is expressed in the olfactory areas during passive exposure to aversive, highly volatile, or biologically significant odorants^7–10^. Oscillations in the beta band are also modulated by learning and beta amplitude in the olfactory bulb (OB) was found to increase with improved performance in a Go/No-Go task^11^. Conversely, in a two alternative choice (TAC) discrimination, OB local field potentials (LFP) were dominated by gamma (40–90 Hz) oscillations during fine odor discrimination^12^. No modulation in the power of OB beta oscillations (15–28 Hz) was shown over learning although beta band activity defined a coherent network between OB and piriform cortex^13^. It was later suggested such differences between studies could be explained by task demand, phase of training, and/or odor set discrepancies^14^.

Relative to the network involved in the generation/expression of beta activity, the only converging data is that beta expression in OB and piriform cortex is strongly impaired if the connection between OB and cortex is disrupted^15,16^. Importantly, most studies using multisite recordings reported that, when expressed, beta oscillations appear across a large network. Rats expressed enhanced beta activity in the OB and both dorsal and ventral parts of the hippocampus during odor sampling in an odor discrimination task^17^. In a conditioned odor aversion task, the aversive behavior was predicted by a strong beta oscillation emerging in a large network including the OB, piriform cortex, orbitofrontal cortex, and basolateral amygdala. This network extended to the infralimbic and insular cortices when the conditioned odor was added to water and stimulated taste during acquisition^18^. Finally, beta coupling between olfactory and motor brain regions was reported in a Go/No-Go task prior to odor-guided movement initiation^19^. These studies converge towards the idea that beta expression goes beyond olfactory areas and suggest it represents widely coherent states^20^.

In an attempt to characterize the conditions of beta expression in a large network, we investigated beta activity in brain areas known to be involved in an olfactory TAC task. These areas included sensory, limbic and sensorimotor regions. Beta expression was analyzed during a simple discrimination, rule transfers, short- and long-term memory recall tests, and reversal tasks. We observed that beta amplitude was correlated with the level of expertise that the animals achieved on the context (odor qualities and reward sides). Notably, we always observed a decrease of beta expression prior to learning of a new discrimination. In addition, during a reward side reversal task, beta expression started high and had to decrease before learning the new contingencies. This suggests beta rhythm could signal a behavioral inflexibility. Finally, when expressed, beta appeared in a large network including olfactory areas and striatum, but neither hippocampus nor cerebellum. Overall, our data suggest that beta rhythm signals a consolidated, inflexible and widely coherent state.

## Results

**Figure 1:**
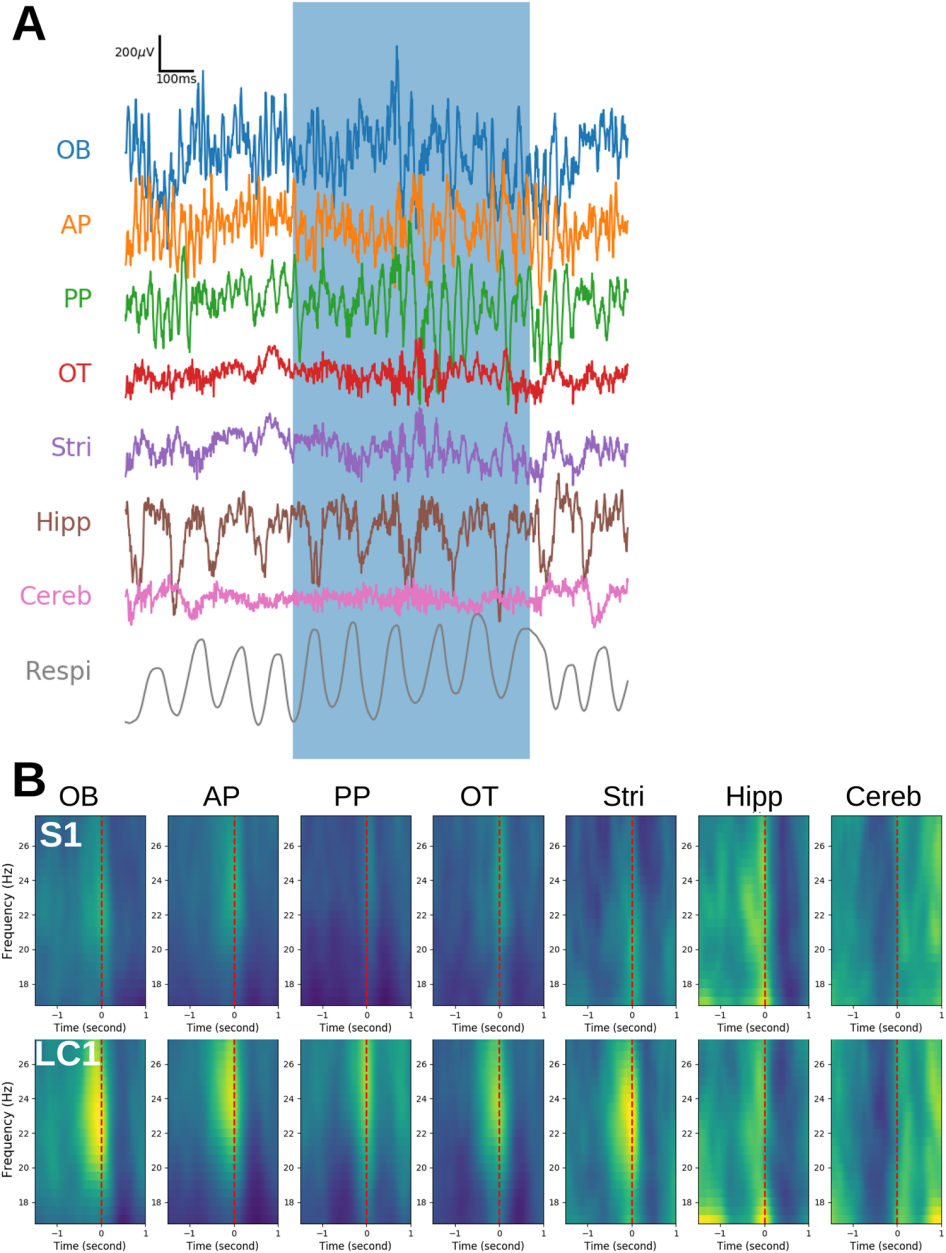
Example of raw recordings and time frequency maps. (**A**) Example of raw signals recorded in olfactory bulb (OB), anterior piriform cortex (AP), posterior piriform cortex (PP), olfactory tubercle (OT), striatum (Stri), dorsal hippocampus CA1 (Hipp), cerebellum (Cereb) and the respiration signal (Respi) of one rat around one trial when the rat has reached the learning criterion of performance. Blue box indicates the presence of the rat in the odor port. In this example, we observe clear beta oscillations (frequency around 25 Hz) close to the end of odor sampling in OB, AP, PP, OT and Stri. (**B**) Average wavelet time frequency maps in the beta frequency range around the end of odor sampling (red dashed lines) for all structures at sessions S1 (upper plots) and LC1 (lower plots) for odor pair P1. Maps are first computed for all trials of a given session and rat, aligned with the end of odor sampling, averaged across trials for each rat and finally averaged across rats. The color scales are independent across structures but identical across sessions for each structure. Warm colors indicate high power. From S1 to LC1, we observe a clear increase of a high power bump around 25 Hz, close to the end of odor sampling, for all structures except Hipp and Cereb.

We trained rats to discriminate odors in a TAC task while recording LFPs in different brain areas (see Table 1 and Material and methods for behavioral paradigm and details of recorded structures). LFPs were collected from 13 animals and Table 1 gives a summary of analyzed regions per animal and session. Raw LFP data (Fig. 1A) and time-frequency maps (Fig. 1B) indicated a strong beta wave at the end of the sampling period during the acquisition of the task (first training session, S1, vs the first session at criterion, LC1, Fig. 1B). In the following sections, we tracked this beta burst (see Methods) and analyzed the different conditions favoring its expression.

**Table 1:** Behavioral paradigm and details of recorded structures and sessions per rat.


Rat #	Structure							Session																		
	OB	AP	PP	OT	Stri	Hipp	Cereb	P1				P2					P3				P1		R1			
								S1	S2	LC0- LC2	LC3	S1	LC0	LC1	LC2	LC3	S1	LC0	LC1	LC2- LC3	T1	T2	S1	LC0- LC1	LC2	LC3
1	X	X		X	X	X		X	X	X	X	X	X	X	X	X	X	X	X	X	X	X				
2		X	X	X	X	X		X	X	X	X		X	X	X	X		X	X	X	X	X				
3	X	X		X	X	X		X	X	X	X		X	X	X	X			X	X	X	X				
4	X	X	X	X	X	X		X	X	X		X	X	X	X	X	X	X	X	X	X	X	X	X	X	X
5	X	X	X	X	X	X	X	X		X	X			X	X	X		X	X	X	X	X	X			
6	X	X	X	X	X	X	X	X		X	X	X	X	X	X	X	X									
7	X	X	X	X	X	X	X	X		X	X		X	X	X	X			X	X	X	X				
8	X	X	X		X	X		X		X	X			X	X	X	X									
9	X	X	X	X	X	X	X	X	X	X	X	X	X	X	X	X	X				X	X	X			
10	X	X	X	X	X	X	X	X	X	X	X	X	X	X	X	X	X	X	X	X	X	X	X	X	X	X
11	X	X	X	X	X	X	X	X	X	X	X			X	X		X				X	X	X	X		
12	X	X	X	X	X	X	X	X	X	X	X	X	X	X	X				X		X	X	X	X	X	X
13	X	X	X	X	X	X	X	X	X	X		X	X	X							X	X	X	X	X	
Total	12	13	11	12	13	13	8	13	9	**13**	11	7	11	**13**	12	10	7	5	**8**	7	11	11	7	**5**	4	3

**Top:** schematic view of the learning protocol across odor pairs (see Material and methods for details). **Bottom:** details of recorded structures (left part of the table) and sessions (right part of the table) for each rat included in the study. The total number of rats recorded for each session is at the bottom of the table, but since not all structures have been recorded in all rats, analyses by structure may include less rats than this total number. OB: olfactory bulb; AP/PP: anterior/posterior piriform cortex; OT: olfactory tubercle; Stri: striatum; Hipp: hippocampus; Cereb: cerebellum. P1, P2, P3 refer to distinct odor pairs. R1 is the reversal of contingencies for P1. Sn is the n^th^ day of training with a given odor pair, LCn is the n^th^ day at criterion (LC0 being the last day before reaching criterion). T1 and T2 are short- and long-term recall tests of odor pair P1.

### Beta activity signals a well-learned discrimination

**Figure 2:**
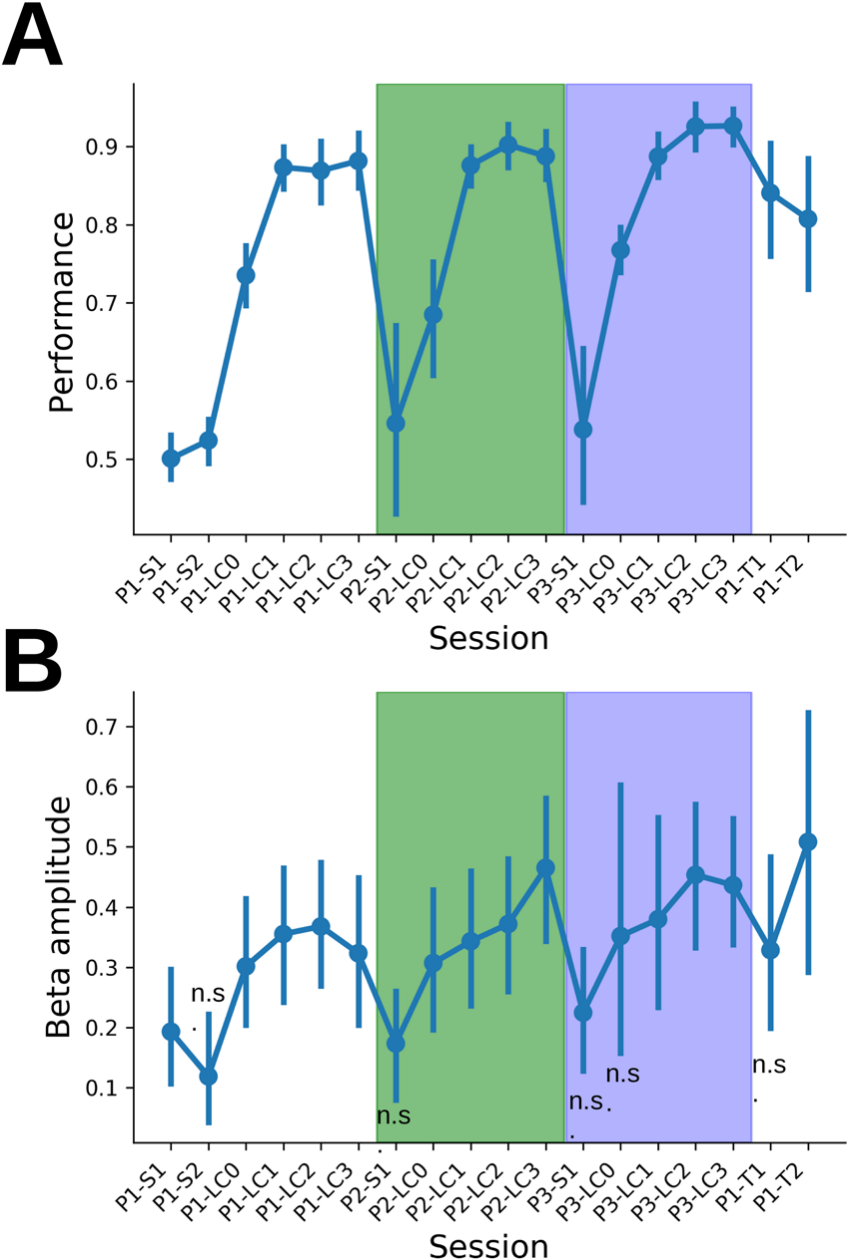
Learning strategy and evolution of beta rhythm across learning. Evolution across experimental sessions of performance (**A**) and AP beta amplitude. (**B**) Points represent the average across 13 rats, error bars show the 95% confidence interval of the mean computed with a bootstrap method. White, blue and green boxes contain sessions of P1, P2 and P3 odor pairs, respectively. For AP beta amplitude, single rat measure is obtained by taking the median across all trials of a given session and statistical analyses have been done using linear mixed models (see Material and methods for details). There is a significant session effect (anova: F(17,11.0) = 13.7, p < 0.001). Individual session comparisons to P1-S1 session show a significant difference for all sessions of all odor pairs except S*x* sessions (t-tests, p < 0.05, only non-significant differences ‘n.s.’ are shown on the graph). The number of rats in each session is given by Table 1.

Figure 2A shows task performance (mean and 95% c.i.) across sessions. For each odor pair, performance started around 0.5 (chance level) and then progressively increased up to 0.9 on average. Note that during learning of the first odor pair (P1), rats acquired both task rules and odor pair discrimination. For the following odor pairs P2 and P3, the discrimination was achieved more rapidly suggesting a transfer of task rules acquired with P1 to the following pairs (number of sessions to reach LC1 (mean + /−sem) for P1: 7.8 + /−1.4 sessions, range 3–19, N = 13 rats; P2: 3.3 + /−0.6 sessions, range 1–9, N = 13 rats; P3: 2.8 + /−0.8 sessions, range 1–7, N = 8 rats).

We tracked the dynamics of beta expression during the different sessions. Figure 2B shows data for the anterior piriform cortex (AP) but the same time course was observed in all structures except the hippocampus (Hipp) and the cerebellum (Cereb) where beta amplitude was near 0 (not shown). During P1 acquisition, beta amplitude increased concomitantly with performance. Remarkably, when a new pair was introduced (P2, rule transfer), beta amplitude dropped (t-test following mixed linear model analysis, see *n.s*. on Fig. 2B indicating sessions where beta amplitude could not be distinguished from first session P1-S1). Then, beta power re-emerged and increased as the animals acquired the new discrimination. This effect was also observed for the third discrimination (P3).

**Figure 3:**
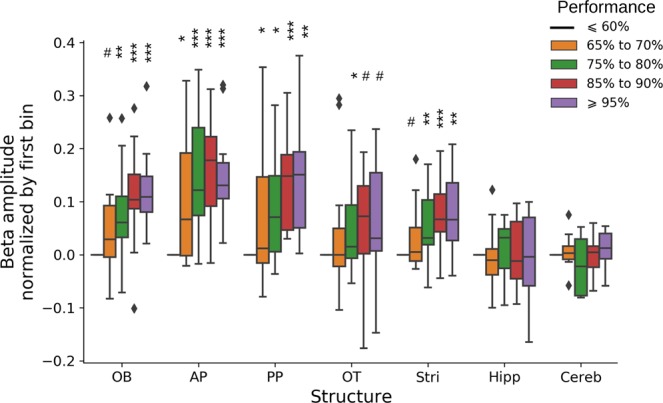
Evolution of beta amplitude during learning of odor pair P1. Evolution of beta amplitude during chunk of 20 trials as a function of performance for all recorded structures. Beta amplitude is computed in chunk of 20 trials (10 consecutive trials per odor) for all sessions of phase P1 (except P1-T1 and P1-T2). Whisker boxes show the distribution across rats of their median beta amplitude in the corresponding performance bin (normalized by the amplitude in the bin of lowest performance, ≤ 60%). Diamonds are single data points which are beyond 1.5 the interquartile range. Linear mixed models have been fitted independently for each structure. Global performance effect was significant for all structures except Hipp and Cereb, and only a trend for OT because of the large variability (anova F-tests: OB: F(4,16.7) = 15.1, p < 0.001; AP: F(4,14.1) = 12.6, p < 0.001; PP: F(4,11.3) = 7.3, p = 0.004; Stri: F(4,13.5) = 5.7, p = 0.007; OT: F(4,9.8) = 2.7, p = 0.096; Hipp: F(4,9.9) = 0.3, p = 0.88; Cereb: F(4,10.5) = 0.2, p = 0.94). For each structure, significance of the difference of each performance bin with performance bin ‘≤60%’ are shown on the graph (t-tests): ^#^p < 0.1, *p < 0.05, **p < 0.01, ***p < 0.001.

Next, we tested more precisely to what extent beta amplitude and performance were correlated during the first acquisition (P1). Figure 3 shows the evolution of beta amplitude in blocks of 20 trials (10 per odor, see Material and Methods), for all recorded structures, as a function of behavioral performance during the block. Data were normalized by the lowest performance block to facilitate comparison across structures. Beta amplitude clearly increased with performance (statistical significance was obtained by fitting data with linear mixed models, see Material and Methods and Fig. 3). This effect was found for all the structures (except for Cereb and Hipp), but, notably, beta amplitude increased to a greater extent and more rapidly in AP (see Fig. 3: larger and quicker change in amplitude in AP as soon as performance was above chance level), closely followed by OB and PP, then by OT and Stri.

Overall, these results suggest that beta oscillations emerge when the rat achieves a good level of discrimination performance and diminishes in parallel with poor performance when the odor pair is changed.

### Beta activity signals a consolidated learning

**Figure 4:**
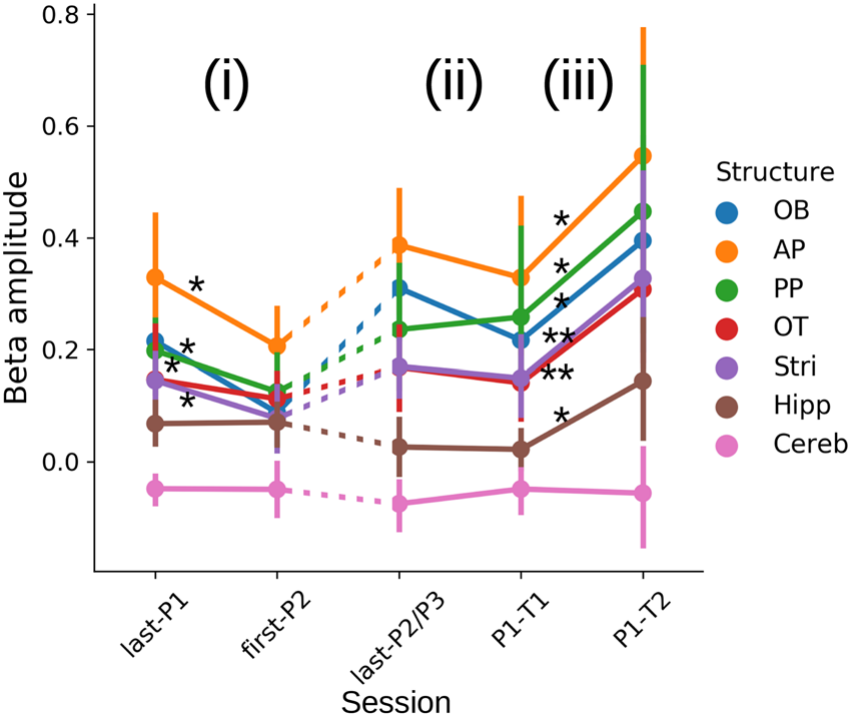
Comparison of beta amplitude during pair transitions. Evolution of beta amplitude for each structure between (i) last LC session of P1 (last-P1) and first session of P2 (first-P2) and (ii) short and long-term test sessions (T1 and T2 of odor pair P1) and the session immediately preceding P1-T1 session (last-P2/P3). As it can be inferred from Table 1, last-P1, first-P2 and last-P2/P3 sessions may correspond to a different level of expertise for each rat. Points represent the average across rats of their median beta amplitude in a given session. For P1-T2, all rats have been pooled whatever their performance. A linear mixed model on data from sessions last-P1 and first-P2 including Structure and Session as random and fixed effects shows significant effect for both Structure (anova: F(6,13.2) = 54.85, p < 0.001) and Session (anova: F(1,12.02) = 6.53, p = 0.025). Structure by structure Wilcoxon tests show that the session effect is present only for OB, AP, PP and Stri (p < 0.05, * on the graph, N = 13 rats). Fitting the same linear mixed model to sessions pre-P1-T1 and P1-T1 showed no statistical difference between sessions (anova: F(1,9.86) = 0.11, p = 0.75). Finally, a significant increase of beta amplitude was found from P1-T1 to P1-T2 (anova, session effect: F(1,9.74) = 13.24, p = 0.005; structure by structure analysis with Wilcoxon paired tests, N = 11 rats, see graph: *p < 0.05 or **p < 0.01).

At this stage, we could not determine whether the marked attenuation of beta amplitude when a new odor pair was introduced was because the odor pair was novel or because of changing the rat’s routine. To clarify this issue, we compared the evolution of beta amplitude in three conditions: (i) when the odor pair changed for a new one (last-P1 → first-P2), (ii) when the odor pair changed but for a previously learned one (last-P2/P3 → P1-T1), (iii) when the odor pair did not change but after 1 month without training (P1-T1 → P1-T2). Results are presented in Fig. 4. First, as already described above for AP (Fig. 2), we found a decrease in beta amplitude when the odor pair changed for a new one in all structures except OT, Hipp and Cereb (i). Conversely, beta amplitude remained stable in all structures when the odor pair changed for a previously learned one (ii). When a known odor pair was presented after a month without any training (iii), beta strongly increased in all structures except in Cereb. Note that not all rats performed well at P1-T2 which led us to test whether there was a correlation between beta amplitude and performance during the P1-T2 session (Supplementary Fig. 1). It is worth noting that animals performing better in P1-T2 indeed exhibited higher beta amplitude, in all structures (except Hipp, Cereb and OT), with the highest correlations for PP, OB, Stri and finally AP.

Overall, these results suggest that beta is expressed in conditions where the rat’s behavior becomes more stable and learning is well-consolidated.

**Figure 5:**
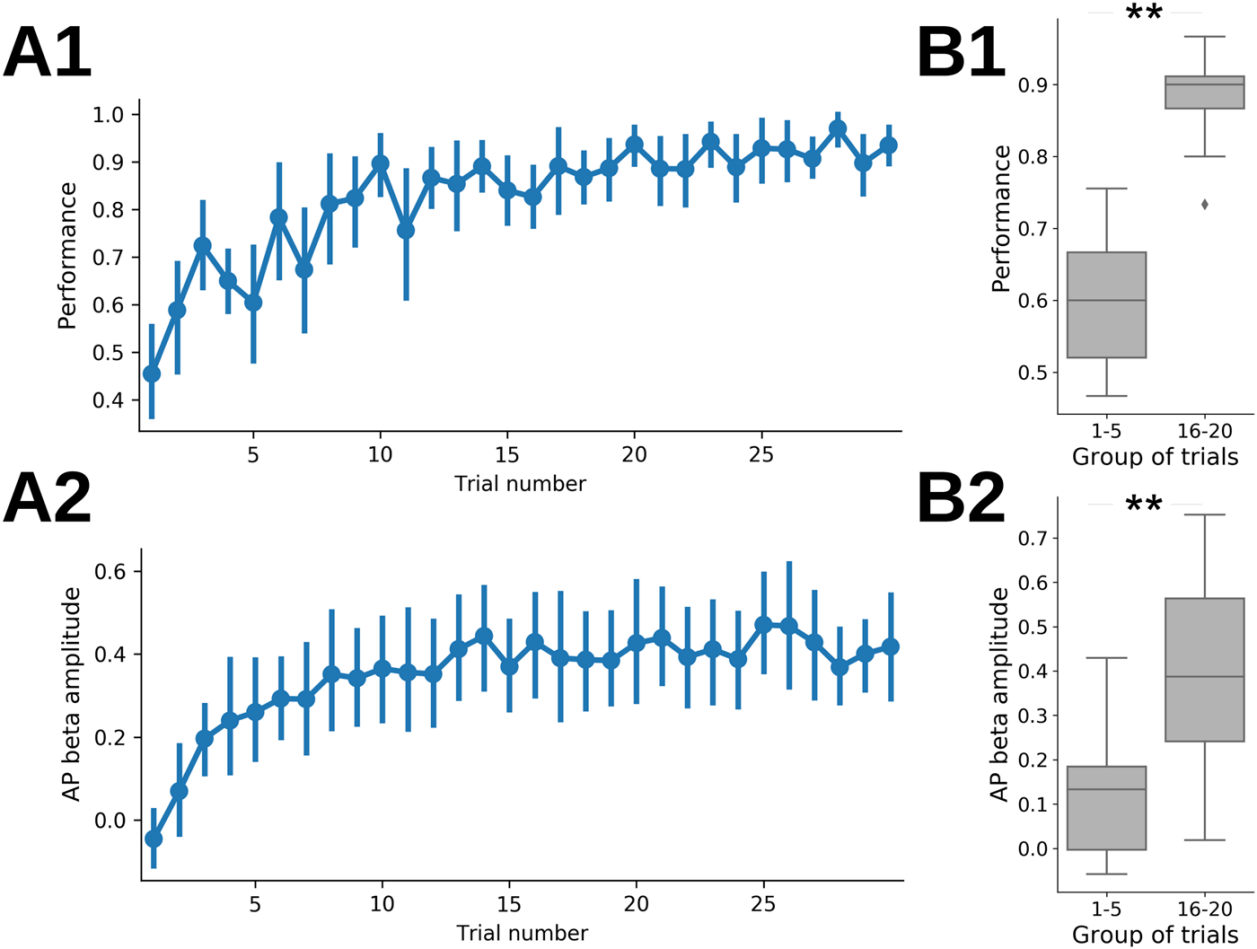
Within session: behavior and beta rhythm dynamics. (**A**) Evolution of performance (A1) and AP beta amplitude (A2) during the first 30 trials for all LC1 to LC3 sessions and odor pairs P1 to P3. For performance, data are first averaged across sessions per rat and then averaged across rats. For beta amplitude, we first computed the median across session for each rat and then plotted the average across rats. In all graphs, error bars are the 95% c.i. of the average across rats. (**B**) Same data as in A, but data from initial trials (1 to 5) and middle trials (16 to 20) were pooled. The group median values are computed for each rat (N = 13 rats) and whisker boxes show the distribution of these median values across rats. Between group comparisons are done using paired-Wilcoxon tests (**p < 0.01). Diamonds are single data points which are beyond 1.5 the interquartile range.

This observation led us to wonder if, once the learning criterion was achieved (LC1 to LC3 sessions), performance and beta amplitude were homogeneous throughout the sessions or whether they increased as the trials accumulated. We thus plotted the average performance for LC1 to LC3 sessions, trial by trial, along with beta amplitude. Results for the first 30 trials are presented in Fig. 5. Performance (Fig. 5A1) and beta amplitude (Fig. 5A2) clearly increased from the beginning of the session to about the 15^th^ trial. This is clearly illustrated by the bar plots in Fig. 5B summarizing the differences in performance (B1) and beta amplitude (B2) between 1–5^th^ trials *vs* 16–20^th^ trials. Such increases were observed individually for each session with a global score > LC whatever the odor pair was (session LC1, LC2, LC3, T1 or T2, odor pair P1, P2, P3 or R1, not shown). Thus, even when well-trained for a given discrimination, performance was low during the first 1–5^th^ trials in parallel with low beta amplitude, both being significantly higher during the 16–20^th^ trials.

Importantly, these results show that the time course of within-session performance and beta amplitude recapitulates inter-sessions’ time course.

### Beta activity markedly attenuates before learning of new contingencies

At this stage, we had explored beta expression in different conditions: first discrimination (P1), rule transfer with a second and third discrimination (P2, P3), retention test of a previously learned pair (T1 and T2). However, we lacked a condition where the odor context was well known but the rules were changed. This was tested during the reversal test (R1).

**Figure 6:**
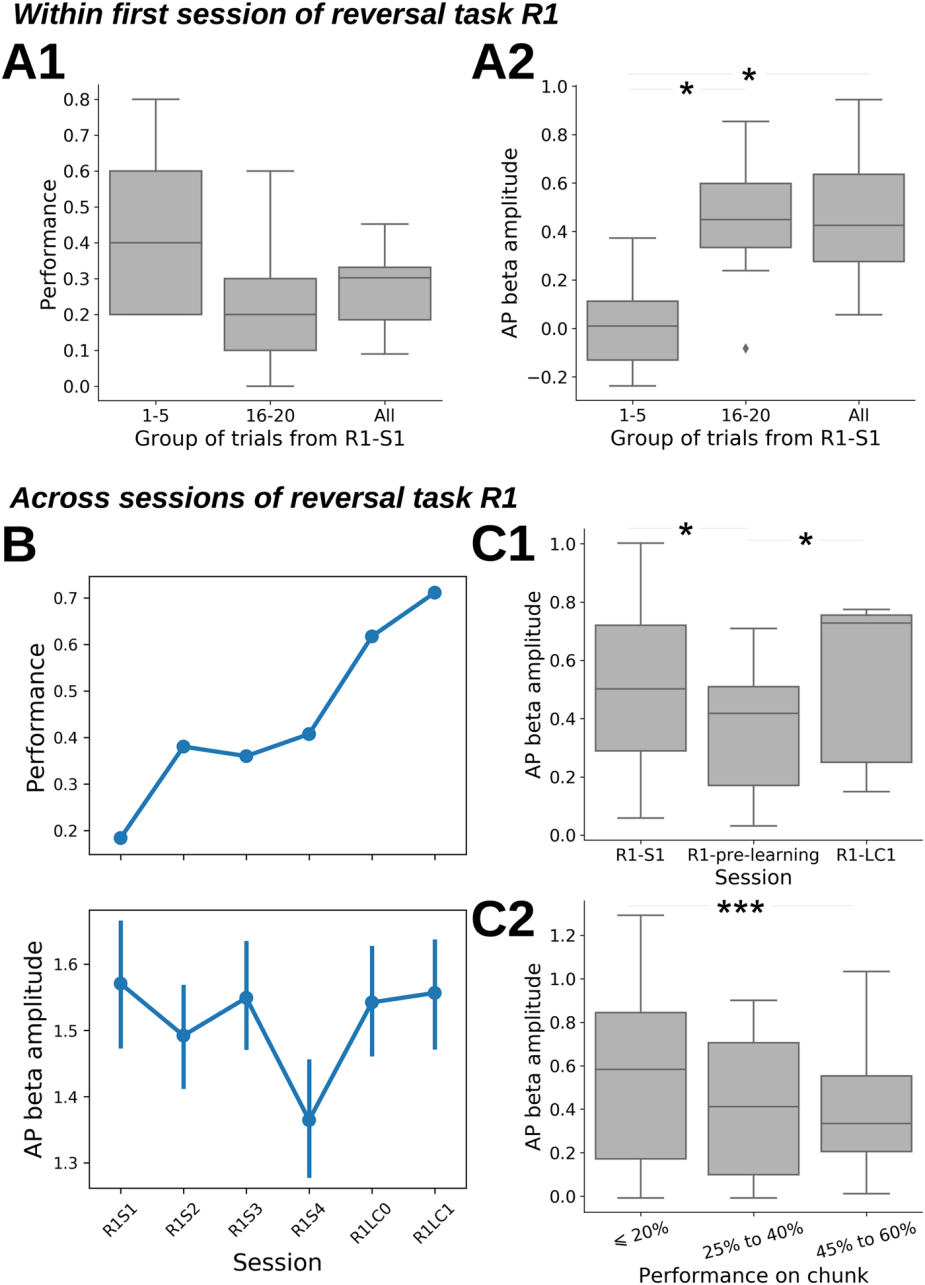
Reversal learning of odor pair P1. (**A**) Performance (A1) and AP beta amplitude (A2) during the first session of reversal of odor pair P1 (R1-S1). Three groups of trials are shown: all trials, trials 1 to 5 and trials 16 to 20. Whisker boxes show the distribution across rats (N = 7) of individual performance or individual median AP beta amplitude. Diamonds are single data points which are beyond 1.5 the interquartile range. Regarding panel A2, a linear mixed model shows the statistically significant increase of AP beta amplitude across bins (anova: F(2,7.05) = 7.74, p = 0.017) confirmed by paired Wilcoxon tests (see graph *p < 0.05, N = 7). In parallel, the performance during trials 16 to 20, and globally over the whole session, is very low (all 7 rats have a session performance below 50%) because rats were still associating odors with previously learned sides (as learned during P1) where they had a maximal performance. (**B**) Example of performance (top) and AP beta amplitude (bottom) evolution across experimental sessions for one rat during reversal learning of odor pair 1 (R1). For AP beta amplitude, points represent the average across trials in each session. Error bars are the 95% c.i. of the average. (**C**) AP beta amplitude changes during reversal learning of odor pair 1 (R1). C1, across sessions**:** R1-pre-learning is the last S*x* session where performance is below 60% (see Methods) and can correspond to a different session for each rat. N = 7 rats for R1-S1 and R1-pre-learning and N = 5 rats for R1-LC1. The first 15 trials from each session are excluded because, as seen in Fig. 5, AP beta amplitude at session start is decorrelated from whole session performance. Whisker boxes are computed as for panel A. A linear mixed model shows a session effect (anova: F(2,5.89) = 10.78, p = 0.017). Session pairs are compared using paired-Wilcoxon tests (*p < 0.05). C2, across trial chunks, as a function of performance within chunk: to cope with change in AP beta amplitude within sessions, data were pooled by chunk of 20 trials (10 per odor, see Methods), for all R1 sessions up to R1-pre-learning, excluding first chunk of each session. A linear mixed model shows a clear performance effect (anova: F(2,10.8) = 15.1, p < 0.001), between bins effect is statistically significant between ‘≤20%’ and ‘40% to 60%’ performance bins (t-test, df = 9.58, t = −3.89, p = 0.003).

Seven animals were tested for reversal, with 5 of them reaching the learning criterion (number of sessions to reach criterion + /−sem: 6.2 + /−0.7, range 4–8, N = 5 rats). Figure 6A shows that performance dropped at the first reversal session (Fig. 6A1). During the 1–5^th^ trials, rats searched randomly for the reward, as they usually did in other sessions and performance was close to chance level 50%. But, after a few trials, even if the odor-side association was reversed, they persisted in using the previously learned odor-side association for P1 which led to a performance below chance level during the 16–20^th^ trials, and more generally for the whole first reversal session. Concomitantly, beta amplitude evolved as in sessions where animals performed well: low in the first 5 trials, higher in the 16–20^th^ and remaining trials (Fig. 6A2).

Figure 6B shows performance (top) and AP beta amplitude (bottom) for an individual rat, during all its reversal sessions. In this example, the animal reached the learning criterion during the 6^th^ session (Fig. 6B top). During the 1^st^ session (R1-S1), the performance was low (the animal persisted in the preceding rule). Then, during the 3 following days (R1-S2 to R1-S4) the rat used its initial random strategy. Starting at R1-LC0 session, the rat started to learn the new reward locations and increased its performance. Notably, beta amplitude (Fig. 6B bottom), was high during the first session (R1-S1) while performance was poor. It decreased until the 4^th^ session (R1-S4), to increase again at LC0 and LC1. This example is not an isolated one as shown by Fig. 6C where data were pooled across rats. Beta amplitude (Fig. 6C1) was analyzed during 3 reversal sessions: R1-S1, R1-pre-learning (the last reversal session with performance < 60%) and R1-LC1. The time course of beta amplitude during these 3 sessions was high at the first session (where performance was low), then decreased (up to R1-pre-learning: a session between R1-S2 and R1-S7 different for each rat) whereas performance was close to random behavior performance, to finally reach the highest value at R1-LC1 when the rat reached again the learning criterion. Another way to illustrate beta dynamics during reversal is to show beta amplitude as a function of performance during trial chunks of all the sessions preceding learning criterion (Fig. 6C2). While animals persisted in using the previously learned odor-side association, resulting in very low performance (≤20%), beta amplitude was high. Then, when performance started to increase to reach chance level (45% to 60%), beta started to decrease in amplitude. This beta amplitude modulation was not restricted to AP but was also clearly seen in Stri and less markedly in OB and OT (see Supplementary Fig. 2).

Overall, these results show that (1) beta expression is not related to task performance since amplitude was very high when performance was very low, and (2) in all cases there was a marked attenuation of beta amplitude before the rat started to achieve a new discrimination.

### Beta activity signals a widely coherent state

**Figure 7:**
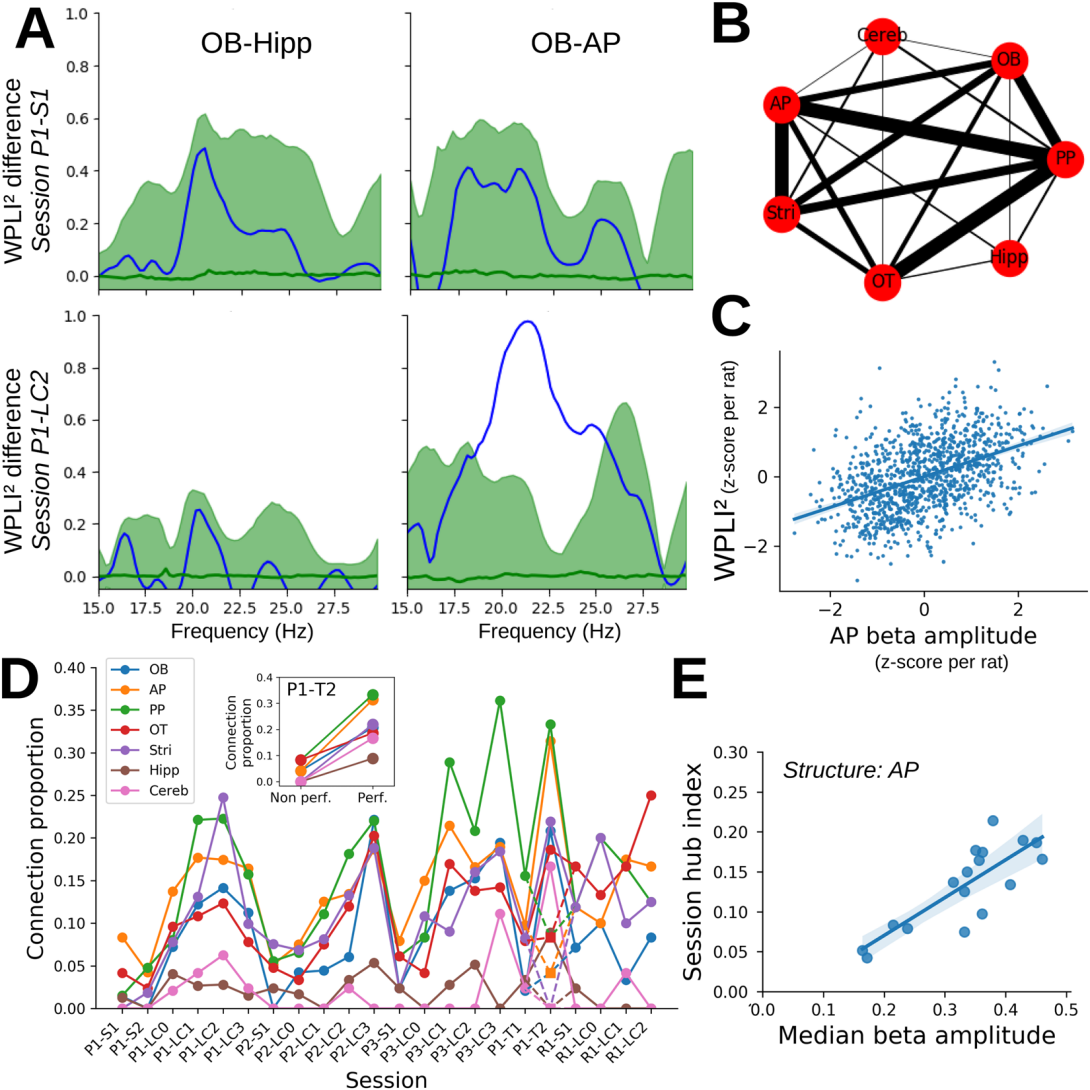
Evolution of network connectivity based on WPLI² index. (**A**) Example of the difference of debiased WPLI² index (blue lines) between time of maximal beta amplitude close to trial end (see Material and Methods for precise determination of this time) versus trial start. Displayed WPLI² indices have been computed between either Hipp and OB areas (left panels) or OB and AP areas (right panels), during the first session of odor pair 1 (P1-S1, upper panels) and the second session at criterion for odor pair 1 (P1-LC2, lower panels). Green areas show the distribution extent (2.5% up to 97.5%), but note that to avoid multiple comparisons across frequencies we compared only maxima of surrogates and real data (see Methods for details). (**B**) Example of beta network based on the proportion of significant links (p_surrogate_ < 0.01) across rats for each structures’ pair (average across sessions LC1 to LC3 of odor pairs P1 to P3). Line widths are proportional to the average proportion of significant connections between two structures. Scale indication: AP-PP average connection proportion is 33%. (**C**) Correlation between AP beta amplitude and WPLI² index (computed at time of maximal beta amplitude and averaged across all structures’ pairs including AP). Each point corresponds to a chunk of 20 trials during learning of pair P1 (see Material and Methods and Fig. 3 for details). Measures have been z-scored for each rat to allow a graphical comparison across rats. A linear mixed model on the original data show there is a strong beta amplitude effect (t-test: df = 12.46, t = 8.99, p < 0.001). Linear regression: slope: 0.21, p < 0.001, R² = 0.28, N = 1092 data chunks. (**D**) Evolution across sessions of the proportion of significant connections for each structure (among all pairs involving the structure and across rats). At P1-T2 session, full, resp. dashed, lines represent data for performer (N = 5), resp. non performer (N = 4) rats during the P1-T2 session (see also inset, and Material and Methods for details). (**E**) Correlation between AP session hub index (proportion of significant connections within one session averaged across rats) and mean AP beta amplitude (median per rat, then averaged across rats). As in panel (D**)**, data from session T2 have been split according to rats’ performance. Spearman rank correlation 0.8, p < 0.001, N = 17 sessions. The Spearman test was significant for all structures except Hipp and Cereb (not shown).

Until now, we have focused mainly on recordings from the AP. However, as can be seen in Fig. 3B, beta increased with learning in all the recorded structures except Hipp and Cereb. In particular, qualitative changes in beta amplitude across sessions were similar for all structures (not shown). Thus we wondered if this concomitant increase in beta amplitude was the signature of a coherent network and if this network was stable across sessions. Thus we computed a functional connectivity index called weighted phase lag index (WPLI², Vinck *et al*.^21^, see Methods). This measure estimates the phase similarity across trials at each frequency between two signals but eliminates all instantaneous interactions that may arise because of volume conduction. In order to focus on the beta rhythm network evoked at the end of odor sampling, we computed the difference of WPLI² between the point of maximal beta amplitude (see Methods for the determination of this point) and the start of odor sampling, in the 17–28 Hz frequency range. An example of this measure from one rat is shown on Fig. 7A (for 2 sessions and 3 structures only, compared with surrogate data in green). In this example, we observed that the WPLI² measure was clearly above surrogate only for the OB-AP structure pair during P1-LC2 session. Based on the comparison between real and surrogate data, we estimated a p-value for each rat and each pair of structures (see Methods). This allowed us to get an overview of the pairs of structures that were most often phase-locked during beta rhythm (an example is shown in Fig. 7B). In these graphs, the width of the lines connecting structures was proportional to the number of rats showing a significant phase-locking between these structures (p_surrogate_ < 0.01). We observed a single beta network including all structures displaying a high beta amplitude (thus Cereb and Hipp appeared only poorly connected to other structures), but also that the piriform cortex (both AP and PP) seemed to represent the hub of this network due to the greatest number of connections.

Next, we investigated changes in this network across sessions. Our main conclusion was that functional connectivity was mainly determined by beta amplitude. Indeed, Fig. 7C shows a strong correlation between WPLI² index measured at the maximum of beta amplitude (without any selection based on statistical significance of the measure) and the average beta amplitude. In a second step, we computed for each session and each structure, the proportion of significant links for each structure (p_surrogate_ < 0.01, Fig. 7D). We clearly observed that this proportion qualitatively followed the beta amplitude across sessions (compare with Fig. 2B). It was also interesting to see that at P1-T2, the WPLI² connectivity was much higher for rats with a good performance compared to rats with a poor performance (Fig. 7D, solid vs dashed line at P1-T2, and inset) which is also in line with the larger beta amplitude we observed for performer rats at P1-T2 (Supplementary Fig. 1). Note that when separating performer and non-performer rats at session P1-T2, two rats were excluded since they started to perform correctly (>80% success over 10 trials) in the middle of the trials considered to compute WPLI² index. Finally, we computed for each session a hub index (proportion of significantly connected structure pairs in the session), and showed it was strongly correlated with the median beta amplitude of the session (Fig. 7E for AP, but result was similar for all structures - Spearman correlation test: correlation >0.75 and p < 0.001 - except for Hipp and Cereb with no detected correlation).

Overall, considering raw measures of WPLI² index (Fig. 7C) or proportion of significantly connected structure pairs (Fig. 7D,E), we found a clear positive correlation between beta amplitude and functional connectivity measured in the beta frequency range. The beta network is thus wide and coherent across all structures displaying beta oscillations.

## Discussion

Although previous studies have investigated beta rhythm during learning, the present study provides several new important findings. First, we showed that expression of beta activity during a TAC is correlated with the expertise level the animal acquired about the context (odor pair + associated reward ports). Second, in a reversal task, we observed that beta oscillatory activity is high while performance being low and the rats persist in applying the previous contingencies. Then beta markedly diminishes before the acquisition of the new odor-side contingencies, suggesting that beta expression could signal an automatic behavior associated with behavioral inflexibility. Third, we evidenced beta oscillatory activity is expressed in a highly coherent network, which may originate from olfactory areas.

Previous studies investigating beta dynamics over learning^11,13,14,16,22,23^ have related beta expression to the level of expertise of the animal for an odor pair. Our data confirm but also substantially extend this observation showing that beta oscillation is not only a reflection of odor learning, but may also be expressed under conditions of well-controlled behavior as learning becomes more consolidated. Indeed, in the first sessions of the reversal task, beta amplitude was still high while performance was low. Even if not rewarded, animals persisted in their behavior. Beta expression is thus concomitant with behavioral inflexibility. However, a key question is whether beta expression induces behavior inflexibility or rather if behavioral inflexibility induces beta expression. Our observation that learning of the new contingencies started after beta decreased (see Fig. 6C2) could be considered as a sign that beta is expressed when a memory is consolidated and that the resulting very stable network prevents behavioral flexibility.

Coherence analysis indicates that, when expressed, beta oscillations appear in a large network including olfactory areas and striatum, but neither hippocampus nor cerebellum. Our recording protocol was initially designed to target brain areas suspected to be involved in olfactomotor control during a TAC task. Thus, in addition to olfactory areas, we hypothesized that somatosensory/sensorimotor areas such as dorsal striatum and cerebellum could be involved. Regarding cerebellum, different reasons may explain why it seemed excluded from the beta network. For example the targeted region ‘Crus I’ might not be involved in this task. Beta range (on which we focused in this study) may not be the best frequency band for communication between cerebellum and other regions^24^. Indeed, oscillatory activity in the theta^25^ or gamma^26^ ranges could rather tune cerebellum with the somatosensory network.

Although previous studies have reported important hippocampal beta activity in the behaving rat^17,27,28^, we did not find hippocampal beta activity in the present study. This disparity could be because of differences in the tasks used and/or electrode location. The electrode was located in dorsal CA1 in our recordings, just below the pyramidal cell layer. However, Vanderwolf (1992) observed that beta wave was much more prominent in the dentate gyrus (DG) than in CA1^27^. More recently it was shown that the laminar profile of beta oscillations exhibits maximum amplitude at the DG hilus, and much less at the fissure^29^. Furthermore, Granger causality analysis suggests that beta activity is driven from the OB to DG, rather than CA1. Overall, these observations strongly suggest that beta oscillations, which are not volume-conducted from DG to CA1, cannot be detected in our experiments with an electrode in dorsal CA1.

If, as shown in these previous studies^27,29^, beta is not volume-conducted from DG to CA1, it could be interesting to challenge the criticism that beta activity could be volume-conducted in most areas of our study. Since we reported a clear positive correlation between beta amplitude and functional connectivity measure (even if WPLI² index is designed to ignore instantaneous interactions, Fig. 7), this possibility cannot be completely ruled out. However, our results are clearly in line with a recent study showing that the link between oscillation amplitude and long-range connectivity is a natural consequence of neural networks^30^.

Our data indicate that the beta network is wide and coherent across all structures displaying beta oscillations. What can be the origin of beta oscillation in this large network? Several points in our data suggest that beta oscillations could originate in the olfactory cortex, at least in our task. First, beta amplitude increases first in AP, then in OB and PP, during the first discrimination. Second, coherence analyses revealed AP and PP as a major hub of the beta network. Vanderwolf (1992) already asked the question of the origin of what he called “fast wave response”. He described an oscillatory activity in the 15–30 Hz range that was elicited by odorants in the DG of freely exploring rats. As this oscillation was never elicited by visual, auditory nor somatosensory stimuli, he concluded that this activity “appears to be related primarily to olfactory input”. We cannot compare this activity to what we recorded in the same frequency range because, in our experiments, it is not odorant-induced but appears with experience. However, we can speculate that beta activity could develop in a large-scale network, when the animal masters the task, from the piriform cortex, then rapidly invading OB, OT and striatum by the large projection network^31^. If it originates in the piriform cortex, which cells and circuits could be involved? A possible circuitry could be the pyramidal cells’ excitation by the cortical association fibers. High-frequency stimulation of association fibers produces a potentiation of the late component of the evoked potential recorded both from the OB and piriform cortex^32^. Interestingly, this phenomenon displays some characteristics of beta oscillation expression: (1) it develops gradually, requiring several daily potentiation treatments to reach maximum amplitude, similarly in our study beta appeared gradually during each discrimination learning; (2) it persisted in latent form for at least 8 days following its apparent decay and could be reinstated by repeated test stimulation. This characteristic is similar to what we observed during long-term testing. Moreover, using optical dye recording in anesthetized rats, it was reported that the late component evoked in the piriform cortex by OB stimulation is larger in animals previously trained on an olfactory task than in naive controls^33^. Here too, this late component seems to share some characteristics with beta expression in our study. As suggested by Stripling and Patneau^34^, the circuit generating the late component could involve a population of pyramidal cells in the deep portion of layer III and in the endopiriform nucleus. Due to their unusual intrinsic properties^35^, these cells could be readily activated by stimulation of OB and/or piriform cortex association fiber system. If we assume that beta oscillations and the late component of electrically evoked potential rely on the same circuits, then we could speculate that beta oscillations evoked at the end of trials in our study may have an olfactory origin.

In conclusion, initiated in a population of olfactory cortex pyramidal cells, beta activity coherently develops across a large network, emerges during learning, when the animal recognizes a well-known context. This could explain why the beta wave appears at the end of odor sampling. In this network, including sensory and task-related regions, beta activity expression seems to be associated with a lack of behavioral flexibility, characterizing an automatized behavior. Thus, more than reflecting the status quo^4^, beta activity could be the signature of a non-flexible network state.

## Materials and Methods

### Experimental procedures

#### Animals

Thirteen adult male Long Evans rats were used (Janvier Laboratories, Le Genest Saint Isle, France; 8 weeks old, ~250–300 g at the start of the experiment). Rats were housed individually in a temperature (22 ± 1 °C) and humidity (55 ± 10%) controlled room and exposed to a 12/12 h light/dark cycle (light onset, 6:00 am). Experiments were conducted during the light period (between 9:00 am and 1:00 pm). Food and water were available *ad libitum* before surgery and during recovery period. During the experiments, food was available *ad libitum* but access to water was restricted to 15–30 min in the experimental cage plus 30 additional minutes in the home cage at 4:00 pm. Rats were handled and weighed daily to control for adaptation to hydric restriction and ensure that their body weight was maintained to at least 80% of their *ad libitum* weight. All the procedures have been performed in accordance with the European Union guidelines and regulations (Directive 2010/63/EU of the European Parliament and of the Council of the European Union regarding the protection of animals used for scientific purposes), they have been approved by the French ethical committee no 055 and the project is referenced by the French Ministry of Research as APAFIS #17088-2018101210568066 v1.

#### Surgery

Anesthesia was induced by an initial dose of a mixture of chloral hydrate and sodium pentobarbital (intraperitoneal; Equithesin 3 ml/kg) and maintained with additional doses as needed. Rats were administered anti-inflammatory and analgesic treatment (subcutaneous; carprofen 2 mg/kg or meloxicam 0.2 mg/kg) immediately after surgery and during several postoperative days if necessary. Rats were daily monitored and allowed to recover for at least 10 days before recording sessions.

Animals were implanted with monopolar stainless steel LFP recording electrodes (diameter: 100 µm, 200–800 kΩ impedance, California Fine Wire) soldered to a copper wire (diameter: 250 µm). Electrodes were positioned stereotactically into the left cortical hemisphere in seven brain areas comprising the olfactory bulb (OB; 5.5 mm posterior to nasofrontal suture, 1.5 mm lateral, ~2 mm deep below the surface of the brain), anterior piriform cortex (AP; 2.76 mm anterior to bregma, 3.5 mm lateral, ~6 mm deep), posterior piriform cortex (PP; 2.4 posterior to bregma, 5.5 mm lateral, ~8 mm deep), olfactory tubercle (OT; 0.24 anterior to bregma, 2.8 mm lateral, ~9 mm deep), dorsal striatum (Stri; 1.08 anterior to bregma 3.5 mm lateral, 3.5 mm deep), dorsal CA1 of the hippocampus (Hipp; 3.8 mm posterior to bregma, 2 mm lateral, ~2.3 mm deep) and in the Crus I region of the cerebellum (Cereb; 12.24 mm posterior to bregma, 3 mm lateral, 1.5 mm deep). Electrophysiological activity was recorded while the electrode was lowered and positioning of the recording electrodes in the AP, PP and OT was determined by the shape of evoked field potentials induced by stimulation of a bipolar OB electrode placed close to the mitral cell layer. The final electrode position was selected where the evoked potentials had the largest amplitude (before shape reversal). The stimulation electrode was then replaced by a monopolar recording electrode in the OB. The depth of the recording electrodes in the OB and Hipp was adjusted using their large multiunit activity, to the level of the mitral cell layer and just below the pyramidal cell layer, respectively. Positioning of the other recording electrode tips (Stri and Cereb) was achieved stereotactically. Each electrode was individually fixed to the skull using dental cement. A reference wire was connected to a skull golden screw located above the posterior portion of the contralateral cortical hemisphere. Two anchor screws were also inserted on the contralateral side to secure the implant. Each electrode was attached to a 32-pin electrode interface board (EIB, NeuraLynx, Inc, USA, ViewPoint France) combined with an omnetics connector and centered on the animal’s head.

Signals were acquired by telemetry using a 32-channel wireless recording system (W32 headstage, TBSI, ViewPoint France). Signals were sampled at 15 kHz, amplified (gain 800×) and recorded *via* an acquisition card (USB-2533, Measurement Computing, Norton, MA). Signals were acquired using custom-made software (Neurolabscope) and stored in a SQL database linked with the signal processing software OpenElectrophy^36^.

#### Experimental apparatus

The apparatus consisted of a whole body customized plethysmograph (diameter: 20 cm, height: 30 cm; EMKA Technologies, France) placed in a homemade sound-attenuating cage^37^. It was divided into two independent airtight chambers: one animal chamber and one reference chamber. The pressure changes due to animal respiration were detected by a differential pressure transducer connected to both chambers (Model dpt, EMKA Technologies, France). The plethysmograph was also equipped with three ports (diameter: 2 cm, depth: 2.5 cm) placed 8 cm above the floor of the apparatus. The central odor port was bordered with two lateral reward ports placed 6 cm on each side. The central port was equipped with a capacitive sensor that allowed nose poke detection and was connected to a homemade olfactometer (with a constant flow rate of 400 mL/min). The entry of the rat nose in the odor port (nose-poke) triggered the odor delivery. Odor diffusion was restricted to the odor port. Constant deodorized air also flowed through the top of the chambers at a constant flow rate of 1100 mL/min. A ventilation pump was connected to the plethysmograph that vacuumed out the equivalent of the air pushed into the chambers at 1500 mL/min (400 mL/min + 1100 mL/min). The two reward ports contained pipettes connected to water pumps. Each reward port was equipped with a capacitive sensor that allowed lick detection. One camera (B/W CMOS PINHOLE camera) was placed in a corner of the cage in order to monitor the animal behavior.

#### Behavioral task

Training initially started with 1) habituation phase to the experimental cage (2 days, 15 min/day) and 2) nose poke training phase (5 to 16 days with animals under hydric restriction) in which the animal learned to poke in the central port (no odor) to trigger water delivery at the left and right reward ports. These two phases were performed before surgery. Two weeks after surgery, rats were trained in a two-alternative choice odor discrimination task TAC^37^. Briefly, the animal had to learn to discriminate between two odors presented in a pseudo-random order. A trial was initiated when the rat poked its nose in the odor port, triggering odor delivery for 2 s. The animal chose the amount of time spent in the odor port. All trial durations were shorter that 2 s and as previously observed in the same task^37^, after learning, rats’ sniffing behavior was stereotyped with a high frequency (7–9 Hz) sniffing from the port approach up to the odor port removal. This was a clear indication that rats sampled the odor as long as they were in the odor port and we thus here confound the time in the odor port with the odor sampling time. Odors were associated to water availability at the left and right reward ports, respectively. The animal had 6 s to make its choice. If the choice was correct, the first lick triggered the delivery of 60 µL of water over 2 s. A minimal 7 s interval was imposed between two trials. Animals performed one session per day with an odor pair. Each session was composed of ∼100 trials and lasted 30 min maximum. To obtain a continuous scoring of individual’s performance within one session, we calculated the percentage of correct choices for blocks of 30 trials. Learning was considered as achieved when the animal reached the learning criterion (LC) of 80% of correct trials on two consecutive blocks on three consecutive days.

#### Behavioral paradigm

The behavioral paradigm was divided into 4 major phases (see Table 1).

**Phase 1- Odor discrimination task acquisition**: The animals were trained as described above using a first pair of odors (P1). The first odor pair to discriminate was the same for all rats. The number of sessions to reach the learning criterion (LC) for this first discrimination was highly variable between rats (mean ± sem: 7.8 ± 1.4 sessions, range 3–19, N = 13 rats). In order to take these inter-individual differences into consideration, the sessions considered for analysis were labeled relative to learning criterion achievement: initial sessions were labeled P1-S1, P1-S2, P1-S3, etc; the session just prior to learning criterion achievement was labeled P1-LC0, and the next 3 sessions where the animals reached learning criterion were labeled P1-LC1, P1-LC2 and P1-LC3. At this stage, rats were considered as expert regarding both the task rules and the discrimination of P1.

**Phase 2- Rule transfer to a new odor set**: the animals were trained to discriminate new odor pairs with pairs P2 and P3 presented in a pseudo-randomized order among rats. The discrimination of the new pairs was acquired more rapidly (mean + /−sem, P2: 3.3 ± 0.6 sessions, range 1–9, N = 13 rats; P3: 2.8 ± 0.8 sessions, range 1–7, N = 8 rats). Indeed rats already knew the rules of the task and could transfer them to new discriminations. The sessions considered for analysis were labeled as for P1. Some rats reached P2-LC0 or P2-LC1 on their first session with P2, the same for odor pair P3 (see Table 1 for details).

**Phase 3- Short- and long-term recall tests**: for the first odor pair learned: the animals were tested for P1 discrimination just after the third odor pair (mean + /−sem, short-term test P1-T1: 11.2 ± 1.2 sessions after the end of P1 acquisition, range: 6–18 sessions, N = 11 rats). Rats were then tested again (long-term test P1-T2) with P1 after a resting period (animals were kept in home cage and were not engaged in any task during this period) of 18.8 ± 1.1 days following P1-T1 (mean ± sem, range: 14–27 days, N = 11 rats). As performance during P1-T2 session was highly variable between rats, animals were split into performers and non-performers for this session (performer rats: performance in trials 11 to 20 ≥ 80%, Supplementary Fig. 1).

**Phase 4- Reversal task**: seven of the rats were trained on a reversal task (R1) in which the first odor pair P1 was used but the association between the reward port side and the odor was reversed, *i.e.* odor A previously associated with the left reward port during P1 training was then combined with the right reward port during the reversal task. The animals took several sessions to reach LC (mean ± sem): R1, 6.4 ± 0.5 sessions, range 5–8, N = 5 rats. Note that 2 rats failed to reach learning criterion, and were included in analysis of the initial part of reversal learning only.

The number of trials per session was 83.0 on average (SD: 13.9, range: 41–103, N = 325 analyzed sessions). These numbers were stable across the learning phases 1–4 (range of the average number of trials across phases: 80.9–87.8 trials per session) and comparable across rats (average number of trials per session and per rat: average 81.5, SD: 7.0, range: 68.9–93, N = 13 rats).

#### Odor sets

Odorants used (and their respective vapor pressures in mmHg at 25 °C, obtained from Chem Spider, ACD/Labs PhysChem Module) were p-cymene (1.7 ± 0.2), 1,8-cineol (1.6 ± 0.3), D-carvone (0.1 ± 0.5), L-carvone (0.1 ± 0.5), ethyl caproate (1.7 ± 0.3) and ethyl heptanoate (0.6 ± 0.4). They were all obtained from Sigma Aldrich (St Louis, MO and Fluka, Germany). Odors were presented in pairs. The first pair (P1, N = 13 rats) animals had to discriminate was p-cymene/1,8-cineol. Two other pairs (P2 and P3) were then used with presentation order pseudo-randomized among animals: D-carvone/L-carvone (P2, N = 13 rats) and ethyl caproate/ethyl heptanoate (P3, N = 12 rats, but 4 did not reach learning criterion). The association between the reward port side (left or right) and the odor was also randomized among animals within an odor pair. Odor delivery was controlled by a custom-made olfactometer. Undiluted odorants were contained in a 10 mL U-shaped Pyrex® tube (VS Technologies, France) filled with odorized microporous polymers. Odorized airflow was sent to the odor port at a constant flow rate of 400 mL/min. All odors were delivered in the central odor port at 10% dilution of the saturated vapor pressure.

### Data analysis

#### Behavioral data

Respiratory signals were acquired and extracted using previously described method^37,38^. Each trial was labeled as “correct” or “incorrect” if animal succeeded or not, respectively. Absence of response was considered as an “incorrect”. Animal performance during a session was represented as the proportion of “correct” trials during the whole session (except for the learning criterion achievement, see *Behavioral task*).

### Electrophysiological signals

#### Time frequency map computation

Spectral content of electrophysiological signals was assessed by computing time-frequency power maps using a standard Morlet wavelet transform (using $\sigma =5\pi$). Signal was previously downsampled at 200 Hz and time frequency maps were computed for the whole session with a frequency resolution of 0.5 Hz in the 17–28 Hz range. The wavelet transform was computed in the spectral domain for faster computation.

#### Artifact detection

Some short and high frequency artifacts in the signal could occur when the rat bumped his head or during other abrupt movements. These were removed according to the following 4-step procedure (done independently for each signal):

1. Computation of the wavelet time frequency map between 90 Hz and 250 Hz (in 20 frequency steps, signals were not downsampled for artifact detection), using $\sigma =3\pi$,
2. Average of the time frequency power across frequencies, and computation of median and median absolute deviation (MAD) across time,
3. Artifact detection threshold set at median + 25*MAD,
4. Detection of artifact periods as epochs where average power was above artifact detection threshold.

All trials with a detected artifact on any electrode in a window of 2.5 seconds starting 1 second before the start of nose-poke were discarded. When computing the increase of connectivity between trial start and time of beta oscillation (Fig. 7), any artifact detected in 2-second window around the start or end of the odor sampling period discarded the trial.

#### Beta amplitude calculation

Session time-frequency maps (averaged across trials) showed a clear power increase over learning in the beta band at the end of the odor sampling period. We thus focused our analysis on this period by computing, for each trial, beta amplitude in windows starting 400 ms before the nose-poke end until 100 ms after the nose-poke end. For each trial we computed raw beta amplitude by (1) at each time point, searching across beta band frequencies for the point of highest amplitude, (2) averaging these amplitudes across time points.

In order to compare beta response across trials, sessions and animals, we normalized raw beta amplitude for each trial by computing its ratio with a baseline. Baseline for a given trial was estimated from a 240s-window centered on nose-poke start. In this window, we computed the beta amplitude (measured as explained above) in non-overlapping periods of 500 ms, excluding other nose-poke periods, licking periods (both period types were removed with an extension of 200 ms at start and end) and artifacts periods (with an extension of 2 seconds at start and end). Trial baseline was then taken as the median of beta amplitudes computed in these periods.

The normalized beta amplitude was bounded by 0 and heavy tailed, thus we finally took the logarithm of the normalized beta amplitude to improve beta amplitude distribution visualization and restore an approximate normal distribution of beta amplitude allowing the use of linear model fitting.

In the following, we will simply use the term *beta amplitude* to refer to the final output of this computation. Note that, an absence of change in raw beta amplitude between baseline and nose-poke end gives a beta amplitude of 0, while positive, resp. negative, values of beta amplitude indicate an increase, resp. decrease, of raw beta amplitude close to nose-poke end relatively to baseline.

While analyzing task acquisition during learning of P1 and R1, we considered our data with a finer grained view than whole day sessions by grouping trials of a single session in blocks of 20 trials, including 10 presentations of each odor as contiguous as possible since odor presentation was random. During each block of trials, we computed the animal performance and median beta amplitude across trials.

#### WPLI connectivity measure and single animal statistical significance

To assess functional connectivity between recorded areas we used the weighted phase lag index^21^ (WPLI). This measure allows detecting signal pairs with a stable preferred phase difference across trials at a given frequency. In particular, two signals displaying high amplitude rhythms in the same frequency range but with unrelated phase differences from trial to trial will here appear disconnected.

WPLI is based on the cross-spectral density between two signals but instantaneous interactions are ignored in order to avoid contamination of the measure by tissue volume conduction. The WPLI index is given by:

$$WPLI=\frac{|E[I({S}_{12})]|}{E[|I({S}_{12})|]}$$

But, in this study, we used the debiased WPLI-square estimator given by:

$$WPL{I}^{2}\equiv \frac{{\sum }_{k=1}^{N}{\sum }_{j\ne k}^{N}I({S}_{12,k})I({S}_{12,j})}{{\sum }_{k=1}^{N}{\sum }_{j\ne k}^{N}|I({S}_{12,k})I({S}_{12,j})|}$$

where *S* is the cross-spectral density and $I$ the imaginary part. Note the cross-spectral densities at each trial have been computed with the wavelet transform at the time points of interest. Since the measure sensitivity was dependent on the number of trials, we always computed the WPLI² index using exactly trials 11 to 40 in each session (without counting trials with a detected artifact). Note that beta amplitude can evolve independently for each recorded structure. However, as shown in the results section, beta rhythm usually occurred in a short time window close to nose-poke end similar for all recorded structures. Thus, for the connectivity analysis, we chose to focus on the *time of maximal beta amplitude* (different for each trial). This time was defined as the time of maximal beta amplitude of the structure average time-frequency map (average of time frequency maps of all structures, except Hipp and Cereb, each map being previously normalized by the median absolute deviation of its corresponding raw signal) in a window around nose-poke end starting 400 ms before (or trial start if the trial was too short) until 100 ms after.

The statistical significance of WPLI² index increase between nose-poke start and time of maximal beta amplitude was then computed for each session and each animal following the lines of Maris and Fries^38,39^:

1. Computation of the debiased WPLI² index at time of maximal beta amplitude,
2. Subtraction of the debiased WPLI² index computed at port entrance (nose-poke start time),
3. Surrogate data: random mixing of cross-spectrum from both groups (nose-poke start times and times of maximal beta amplitude) and computation of the difference of debiased WPLI² index between them; repeated 2000 times,
4. Position of the max of WPLI² difference in 17–28 Hz range from real data relatively to the distribution of maxima in the same range from surrogate data. The p-value (for one animal during one session) is finally given by the fraction of surrogate maxima above the real data maximum.

#### Software

Behavioral and electrophysiological raw data were stored in an SQL database linked with the signal processing software OpenElectrophy^36^. All analyses were then made using custom-made scripts written in Python 2.7 with use of toolboxes SciPy 1.0.0, Pandas 0.22.0 and StatsModels 0.9.0^40^. Mixed-model statistical analyses were performed with R 3.4.4 using *afex* 0.25-1^41^. All scripts and data at any stage of analysis are available upon request.

#### Statistical analyses

We had to deal with different issues for statistical analyses: heterogeneity of effects across rats, and heterogeneity in the number of recorded structures and conditions (missing values). For these reasons, all results on beta amplitude have been analyzed using linear mixed models (with the MixedLM class from the Python StatsModels package or the *mixed* function from R *afex* package) grouping data per rat. For each figure graph where we made a linear mixed model, the fixed and random (per rat) effects included the x-axis variable (Session or Block of trials) and the intercept. Interaction between variables were not found significant and thus not included neither in fixed nor in random effects. Fixed-effect significance was assessed with F-tests (*anova* function from *afex*, using type-II ANOVA), the degrees of freedom being approximated using the Satterthwhaite approximation. For a given fixed-effect, single category significance was assessed using a t-test (reported p-values were given by the *afex* “summary” function after fitting the linear mixed model). Unless otherwise stated, fixed effects with p < 0.05 were considered as statistically significant. In all cases, visual inspection of residual plots did not reveal any obvious deviations from homoscedasticity or normality.

Importantly, beta amplitude has been shown to depend on odor quality^9^. In addition our rats did not explore equally both reward sides during training but were mostly biased towards the right side (independently of the associated odor). Thus while analyzing our data we systematically checked the odor and reward side effects by adding them, one at a time, in our linear mixed models both as fixed and random effects. The only substantial effect we found was larger beta amplitude for the biased side (right side for 12 out 13 rats and left side for one rat) only for odor pair P1 in sessions LC0 to LC3. No similar effect was present for other odor pairs P2/P3, neither for tests T1/T2, nor reversal learning R1. Thus data here have been pooled across odors/reward sides.

When specific comparisons between subsets of data points were needed for posthoc analysis, we used Wilcoxon or rank sum tests (for paired or unpaired data as appropriate) on data aggregated per rat (using the median values in each condition).

When many data points from different sessions or blocks of trials are shown, each point was computed as the average across rats of beta amplitude median value or *performance*. Error bars are the 95% confidence interval (c.i.) of these averages obtained using a bootstrapping method (N = 1000 bootstraps). Additionally, we plotted these averages linked across x-axis values with colored lines in order to clarify the visualization (in particular when many structures are shown simultaneously), however, the reader must keep in mind that as shown in Table 1, not all rats have been recorded in all sessions.

## Supplementary information

Supplementary figures

## Data Availability

All data and scripts at any stage of analysis are available upon reasonable requests from Nicolas Fourcaud-Trocmé (nicolas.fourcaud-trocme@cnrs.fr).
