# Supplementary material for: High beta rhythm amplitude in olfactory learning signs a well-consolidated and non-flexible behavioral state
Source: Sci Rep. 2019 Dec 30;9:20259. doi: 10.1038/s41598-019-56340-y (PMC6937317; doi:10.1038/s41598-019-56340-y)
Supplement: Supplementary file 1 — Supplementary figures [file 41598_2019_56340_MOESM1_ESM.pdf]

Supplementary figures for article:

High beta rhythm amplitude in olfactory learning signs a well-consolidated and non-flexible behavioral state

Fourcaud-Trocmé Nicolas[#1]*, Lefèvre Laura[#2], Garcia Samuel[1], Messaoudi Belkacem[1] and Buonviso Nathalie[1].

([#] contributed equally)

1 Lyon Neuroscience Research Center, Inserm U 1028, CNRS UMR 5292, University Lyon 1, Lyon 69366, France

2 Medical Research Council Brain Network Dynamics Unit, University of Oxford, OX1 3TH Oxford, United Kingdom

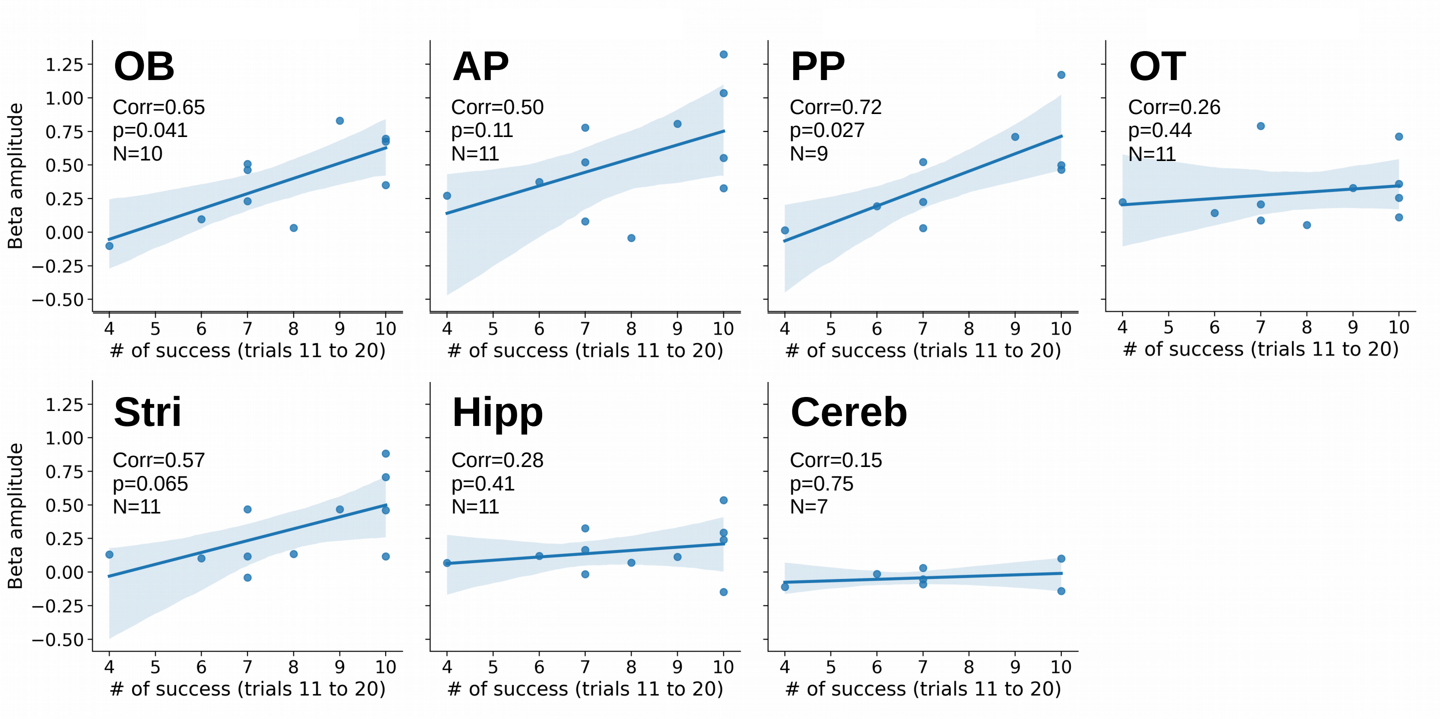

Supplementary Figure 1

Supplementary Figure 1: Beta amplitude as a function of performance during long-term recall P1-T2. Correlation between beta amplitude and number of success for each structure during the P1-T2 session. To minimize relearning effects and ensure context recognition, successes and median beta amplitudes are analyzed from trials 11 to 20. Lines are linear fits with shaded areas being the 95% confidence interval of the slopes. Statistical significance of the correlations have been tested with the Spearman rank correlation test, results are on each graph.

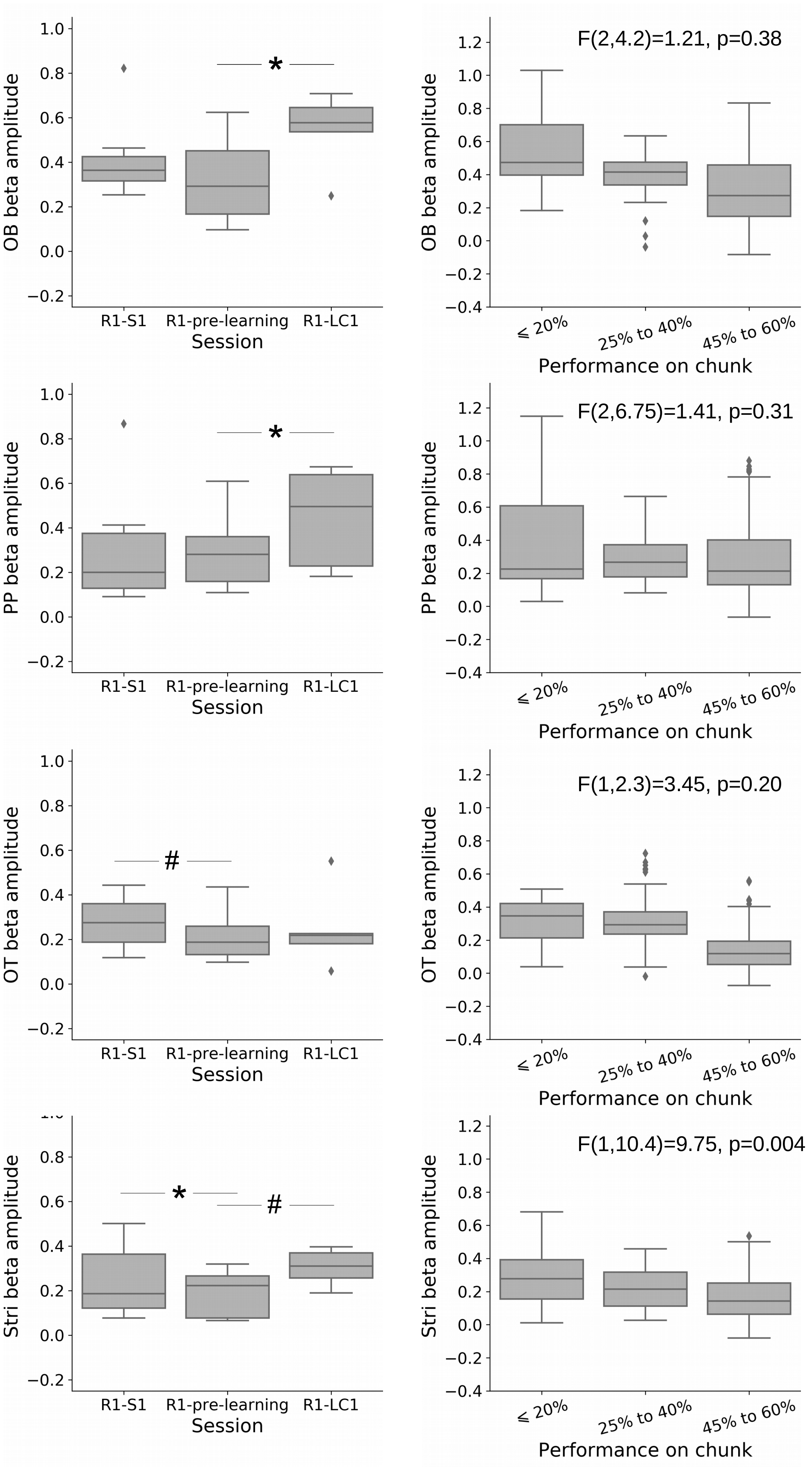

Supplementary Figure 2

Supplementary Figure 2: beta amplitude during reversal learning of odor P1 per structure. Same graphs as in Fig.6C1 (**left**, beta amplitude in sessions R1-S1, R1-prelearning and R1-LC1) and Fig.6C2 (**right**, beta amplitude in chunk of trials grouped by bins of chunk performance, for sessions from R1-S1 to R1-prelearning) for structures OB, PP, OT and Stri (data for Hipp and Cereb are flat, not shown). Difference between sessions in the left panels have been tested with paired-Wilcoxon tests (N = 7 rats between R1-S1 and R1-prelearning; N = 5 rats between R1-prelearning and R1-LC1), results are on the graph: $^{\#}$ $p<0.1$, * $p<0.05$. Performance bin effect on the right panels has been tested with linear mixed models and anova F-tests (results are written on each panel). A separate analysis, using as model fixed effect the chunk performance (continuous variable) instead of the chunk performance bin (categorical variable), reveals a significant decrease of beta amplitude with performance for OB, Stri and a trend for OT (anova: OB: $F(1,5.16)=7.96$, $p=0.036$; Stri: $F(1,6.05)=15.96$, $p=0.007$; OT: $F(1,3.08)=6.76$, $p=0.078$).
